# Endoscopic subserosal dissection combined with the pocket-creation method for the treatment of exophytic gastric gastrointestinal stromal tumor

**DOI:** 10.1055/a-2686-8077

**Published:** 2025-09-18

**Authors:** Guangrong Wang, Jingjing Yao, Yang Liu, Wenwen Hou, Feifei Zhang, Jing Wang, Jindong Fu

**Affiliations:** 1 549615 Department of Gastroenterology, Rizhao Peopleʼs Hospital, Rizhao, China

**Fig. 1:**
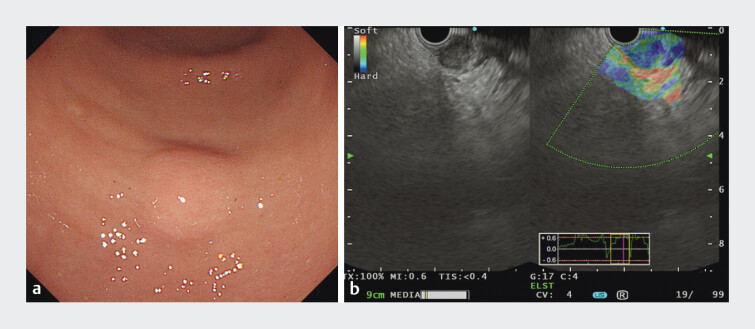
**a** A submucosal tumor at the junction of the gastric antrum and body on the greater curvature, measuring approximately 1.5 cm × 2.0 cm, with a smooth surface; **b** EUS showed a hypoechoic mass originating from the muscularis propria, with clear boundaries and indicating a hard texture, and color Doppler flow imaging revealed no significant blood flow signals.

**Fig. 2:**
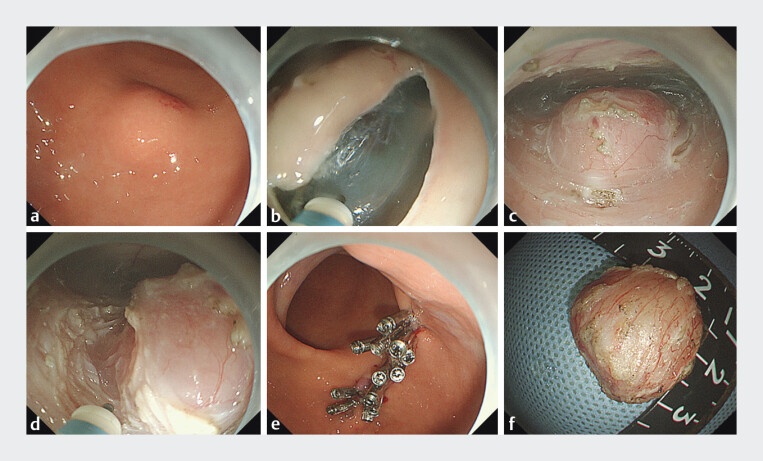
Images of ESSD combined with the PCM. **a** Endoscopic images of the lesion. **b** Transverse mucosal incision on one side of the lesion; **c** Submucosal dissection to create a “pocket-like” tunnel. **d** Circumferential incision of the muscle layer of the tumor, subserous injection to strip the tumor and the subserous layer. **e** Wound closure using preserved mucosal layer and metallic clips. **f** The appearance of the complete tumor.

**Fig. 3:**
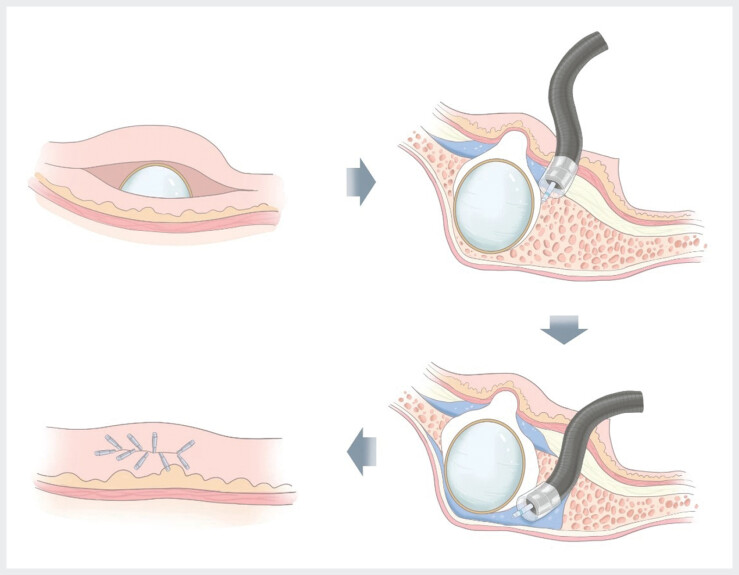
Surgical mode diagram of ESSD combined with the PCM. Create a transverse incision, submucosal injection to strip the submucosa and the surface of the tumor, forming a “pocket” tunnel; circumferential incision of the muscle layer of the tumor; subserous injection to strip the tumor and the subserous layer; and use the retained mucosa to close the wound.

A 72-year-old female patient presented with abdominal pain. Gastroscopy revealed a submucosal tumor at the junction of the gastric antrum and body on the greater curvature, measuring approximately 1.5 cm × 2.0 cm, with a smooth surface ( Fig. 1 **a** ). Endoscopic ultrasound showed a hypoechoic mass originating from the muscularis propria, with clear boundaries. Elastography indicated a hard texture, and color Doppler flow imaging revealed no significant blood flow signals ( Fig. 1 **b** ). The lesion was diagnosed as an exophytic gastric gastrointestinal stromal tumor (GIST). Enhanced abdominal CT confirmed no metastasis. After obtaining informed consent, we performed endoscopic subserosal dissection (ESSD) combined with the pocket-creation method (PCM) ( Fig. 2 , Fig. 3 , Video 1 ). First, a transverse mucosal incision was made on one side of the lesion. Then, a submucosal injection of a mixture (saline + methylene blue + epinephrine) is performed to separate the mucosa from the tumor surface, creating a “pocket-like” tunnel that fully exposed the tumor and provided an optimal surgical field. Next, the tumor muscle layer was then incised circumferentially, the mixture was injected subserosally several times, the base of the tumor was carefully peeled off along the subserosal layer, and the defect was closed with a metal clip on the retained mucosal layer. Postoperative histopathology confirmed a gastric GIST with negative resection margins (1.8 cm × 1.8 cm × 1.3 cm), mitotic count <5/5 mm ^2^ (very low risk), and WHO prognostic group 1.

Endoscopic subserosal dissection combined with the pocket-creation method for the treatment of exophytic gastric gastrointestinal stromal tumor.Video 1

GISTs are common mesenchymal neoplasms of the gastrointestinal tract, requiring treatment that balances radicality and minimal invasiveness. While traditional endoscopic full-thickness resection (EFTR) is effective, it carries a high risk of intentional perforation. In this case, the combined ESSD and the PCM demonstrated the following advantages. First, the “pocket-like” tunnel provided optimal visualization and operative space, minimizing collateral tissue damage. Second, preservation of mucosal and serosal integrity reduced postoperative risks of delayed bleeding, perforation, and intra-abdominal infection. Third, the prevention of gastric content leakage into the peritoneal cavity avoided sudden increases in intra-abdominal pressure, ensuring anesthesia safety. ESSD and the PCM offer significant clinical value for exophytic gastric GISTs and represent a safe, effective minimally invasive treatment strategy.

Endoscopy_UCTN_Code_TTT_1AO_2AG_3AZ

